# Aplastic anemia severity and IL-6 and IL-8 blood levels

**DOI:** 10.15190/d.2022.16

**Published:** 2022-12-31

**Authors:** Sharvan Kumar Bhargawa, Anurag Singh, Geeta Yadav, Rashmi Kushwaha, Shailendra Prasad Verma, Anil Kumar Tripathi, Uma Shankar Singh

**Affiliations:** ^1^ Department of Pathology, King George’s Medical University, Lucknow, India; ^2^ Department of Clinical Hematology, King George’s Medical University, Lucknow, India

**Keywords:** Aplastic anemia, disease severity, interleukin-6, interleukin-8.

## Abstract

INTRODUCTION AND AIMS: Aplastic anemia is a rare, fatal bone marrow disorder that is presumed to be an autoimmune-mediated illness that actively destroys haematopoietic cells through a T helper type-1 cell response. Different cell types in the bone marrow and peripheral circulation produce chemokines, such as interleukin-6 (IL-6) and interleukin-8 (IL-8). The myelopoiesis that is profoundly impaired in aplastic anemia may be inhibited by these two, as critical and powerful inhibitors. Therefore, it is conceivable that their ongoing overproduction may contribute to aplastic anemia. We performed a quantitative enzyme-linked immunosorbent assay on the peripheral blood plasma to reveal the levels of IL-6 and IL-8 and their correlation to aplastic anaemia. MATERIALS AND METHODS: A total of 80 cases of aplastic anemia were included in this study, diagnosed according to the criteria laid down by the International Agranulocytosis and Aplastic Anemia study group. A total of 10 healthy individuals served as controls in this study. With the help of a commercial ELISA kit and the instructions from the kit's maker, the levels of IL-6 and IL-8 were measured in a quantitative way. RESULTS: Mean serum IL-6 and IL-8 levels in cases were 283.28±220.27 and 122.56±97.79 pg/ml, respectively, as compared to 7.52±1.43 and 3.42±1.73 pg/ml levels in controls. Statistically, mean IL-6, as well as IL-8 levels, were significantly higher in aplastic anemia patients than in controls (p< 0.001). Levels of interleukins were also assessed in relation to the severity of the disease. Patients with very severe aplastic anaemia had significantly higher mean IL-6 and IL-8 levels (516.71±36.73 and 220.50±23.45 pg/ml, respectively), followed by severe aplastic anaemia (198.84±150.39 and 89.82±77.18 pg/ml, respectively) and non-severe aplastic anaemia (26.71±33.40 and 10.29±2.63 pg/ml, respectively) (p<0.001). CONCLUSION: Blood serum levels of IL-6 and IL-8 were increased in aplastic anemia and showed a correlation with the severity of the disease. Hence, IL-6 and IL-8 may play an important role in the immune-mediated pathophysiology of aplastic anemia and their increasing levels are giving alarming signals for timely implementation of the appropriate treatment regimen to stop further progression of the disease. Additional studies are required in order to further investigate the exact involvement and role of IL-6 and IL-8 in aplastic anemia.

## INTRODUCTION

Aplastic anemia is a catastrophic bone marrow failure illness. Aplastic anemia is primarily caused by a deficiency of stem cells and their granulocytic, erythroid, and megakaryocytic progenitors in the bone marrow. Pancytopenia, reticulocytopenia, hypocellularity of the bone marrow, and a reduction in hematopoietic stem cells are its defining features^1^_._ In 1904, Vaquez and Aubertin named the illness aplastic anemia^2^_._ Patients with aplastic anemia often present with symptoms of anemia, purpura, hemorrhage, and, less frequently, infections, leading to medical evaluation. Aplastic anemia is more common in developing countries^3^_._The incidence of aplastic anemia is 2 to 3-fold higher in Asia than in western countries^4^_._ Approximately 80% to 85% of cases of aplastic anemia are acquired, and another 15-20% are inherited^5^_._ Aplastic anemia may range in severity from moderate, persistent pancytopenia to complete haematological collapse.

The modified Camitta criteria classify aplastic anemia into three categories on the basis of bone marrow cellularity, absolute neutrophil count, platelet count, and reticulocyte count^6^_._ Hematopoiesis is controlled by different growth factors, cytokines, and chemokines in an autocrine or paracrine manner, produced by marrow stromal cells, regulatory cells, and stem cells^7^_._ Interleukins are a diverse group of cytokines with immunoregulatory functions that include cell proliferation, maturation, adhesion, and migration. Interleukin-6 (IL-6) has a broad spectrum of biological activities, including the differentiation of B-cells and T-cells as well as the activation of macrophages and T-cells^8^_._ Interleukin-8 (IL-8) is a pleiotropic prototype chemokine that is chiefly produced by fibroblasts and macrophages in the bone marrow and in the peripheral blood by neutrophils and T-cells. IL-6 and IL-8 are both potent inhibitors of myelopoiesis and are critical for acute inflammation and tissue damage^9^_._As a result, it is assumed that persistently increased IL-6 and IL-8 productivity may have the potential to cause immune-mediated bone marrow failure in aplastic anemia. In the present study, we evaluated the various haematological parameters and levels of blood IL-6 and IL-8 in aplastic anemia as well as their relationship with the severity of illness.

## MATERIALS AND METHODS

This prospective descriptive study was performed between June 2018 and July 2019, after approval by the Ethics Committee of the King George’s Medical University, Lucknow (No.801/Ethics/R.Cell-18), on May 21, 2018. The International Agranulocytosis and Aplastic Anemia Study Group^10^ criteria were used to diagnose a total of 80 cases of aplastic anemia (45 men and 35 women). The study was carried out at King George's Medical University, Lucknow, a tertiary care hospital in the northern Indian state of Uttar Pradesh, in the Departments of Pathology and Clinical Hematology. Before treatment, all patients had a complete blood count, a peripheral blood and bone marrow smears examination, and serum levels of IL-6 and IL-8.

Hypocellular bone marrow (≤ 25%) on trephine biopsy, without fibrosis or neoplastic infiltration, and at least two of the following were required for case selection: Hemoglobin less than 10gm/dl, platelet count less than 50x10^9^/L and neutrophil count less than 1.5x10^9^/L. It was considered very severe aplastic anemia (VSAA) when the above criteria were fulfilled with an absolute neutrophil count of less than 0.2x10^9^/L and severe aplastic anemia (SAA) when the absolute neutrophil count was less than 0.5x10^9^/L. The remaining individuals were characterized as having non-severe aplastic anemia (NSAA). The research only included participants who provided their written consent. Patients with marrow aplasia caused by chemotherapy, those younger than 35 years with hereditary bone marrow failure syndromes determined by physical examination and chromosomal fragility tests using diepoxybutane, and those who had not provided written informed consent were excluded from the study. The cases of Paroxysmal Nocturnal Hemoglobinuria (PNH) were also excluded from the study by the immunophenotypic determination of clones. Ten healthy individuals were selected for study as controls, and peripheral blood samples were taken in order to estimate the levels of IL-6 and IL-8.

For the collection of samples, informed written consent was obtained from patients and, in the case of children, their legal guardians. In each sterile EDTA and plan serum vial (NOVAC POLYMED), four millilitres of blood were drawn under aseptic circumstances from a peripheral vein. A peripheral blood smear analysis and complete blood count were performed. Each vial of plane blood was centrifuged at 4000 rpm for 10 minutes. Until the IL-6, and IL-8 levels were analysed, serum was extracted and kept at -60 degrees Celsius. According to the manufacturer's instructions, a commercial ELISA kit (Elabscience) was used to quantitatively estimate the levels of IL-6 and IL-8. The findings were represented as the concentration in pg/ml. Under strict aseptic conditions, bone marrow aspiration and biopsy were done from the posterior superior iliac spine in all patients under local anaesthesia using 2% lignocaine solution.

The data were statistically analysed using SPSS (Statistical Package for Social Science) version 21.0 and presented as a percentage, mean ± standard deviation, and P-value. Statistically significant was defined as a p-value of <0.05 or lower.

## RESULTS

A total of 80 cases (45 males and 35 females) of aplastic anemia were included in this study, having a male-female ratio of 1.28:1, along with 10 healthy individuals (5 males and 5 females) as controls. The ages of the cases ranged from 1 to 78 years, while the age range in the control group was from 6 to 53 years. There was no statistically significant correlation seen between aplastic anemia patients and controls for age and gender distribution (p>0.05).

The mean age of NSAA, SAA, and VSAA patients were 21.83±18.73, 18.42± 13.85, and 23.90±17.39 years, respectively. Statistically, there was no significant difference observed in the mean age of patients with different severity grades of aplastic anemia (p = 0.421). The majority of NSAA (66.7%) and VSAA (58.1%) patients were males, while the majority of SAA (51.6%) patients were females. However, the difference among sub-groups of aplastic anemia was not significant statistically (p = 0.446).

As compared to control patients, aplastic anemia patients had statistically significantly lower mean hemoglobin (Hb) (p<0.001), white blood cell count (WBC) (p<0.001), neutrophil percentage (p<0.001), platelet count (p<0.001), red blood cell (RBC) count (p<0.001) and absolute neutrophil count (ANC) (p<0.001) values, whereas aplastic anemia patients had statistically significantly higher mean lymphocyte percentage (p <0.001), mean corpuscular volume (MCV) (p<0.001) and mean corpuscular hemoglobin concentration (MCHC) (p<0.001) as compared to control patients. Statistically, there was no significant difference observed between the two groups with respect to the mean percentage of eosinophils (p = 0.253), monocytes (p = 0.076) and mean corpuscular hemoglobin (MCH) (p = 0.662).

Statistically, no significant difference was observed among different severity grades for mean hemoglobin (p = 0.570), percentage of eosinophils (p = 0.334), monocytes (p = 0.295), RBC count (p = 0.419), MCV (p = 0.680) and MCHC (p = 0.980) values. From NSAA to VSAA, mean WBC count, neutrophil percentage, platelet count, and ANC decreased significantly (p<0.05), whereas mean lymphocyte count increased significantly (p<0.001).

Statistically, there was no significant difference seen among different severity grades for the percentage of erythroid cells (p = 0.094), lymphocytes (p = 0.177) and plasma cells (p = 0.064) present in bone marrow aspiration smears. However, the mean myeloid cell percentage was 19.44±4.37, 15.19±6.87 and 14.68±7.50 & mean marrow cellularity percentages were 22.06±1.95, 19.23±2.14 and 13.87±2.16 respectively in NSAA, SAA, and VSAA, which displayed a decremental trend as the severity of aplastic anemia increased (p<0.05).In comparison to mean serum IL-6 (7.52±1.43) and IL-8 (3.42±1.73) pg/ml levels in controls, cases had significantly higher mean IL-6 and IL-8 levels 283.28±220.27 and 122.56±97.79 pg/ml, respectively (p<0.001) (Table 1).

**Table 1:** Comparison of IL-6 and IL-8 levels between cases and controls

Parameter	Cases (n=80)	Cases (n=80)	Controls (n=10)	Controls (n=10)	Statistical significance
	Mean	SD	Mean	SD	p value
IL-6 (pg/ml)	283.28	220.27	7.52	1.43	<0.001
IL-8 (pg/ml)	122.56	97.79	3.42	1.73	<0.001

Mean IL-6 and IL-8 levels in the NSAA, SAA, and VSAA groups were 26.71±33.40 and 10.29±2.63 pg/ml, 198.84±150.39 and 89.82±77.18 pg/ml, and 516.71±36.73 and 220.50±23.45 pg/ml respectively, with a significant incremental trend with increasing aplastic anemia severity (p<0.001) (Table 2).

**Table 2:** Comparison of IL-6 and IL-8 mean levels in relation to different severity strata of aplastic anemia

Parameter	Non-severe (n=18)	Non-severe (n=18)	Severe (n=31)	Severe (n=31)	Very severe (n=31)	Very severe (n=31)	Statistical significance
	Mean	SD	Mean	SD	Mean	SD	p value
IL-6 (pg/ml)	26.71	33.40	198.84	150.39	516.71	36.73	<0.001
IL-8 (pg/ml)	10.29	2.63	89.82	77.18	220.50	23.45	<0.001

## DISCUSSION

In the present study, the age of aplastic anemia patients ranged from 1–78 years, whereas that of controls ranged from 6–53 years. The mean age of patients was 21.31±16.41 years in aplastic anemia patients compared to 28.00±15.72 years in control patients. Male predominance was also seen in our study, with a male to female ratio of 1.28:1, as in previous studies^11-13^_._ In the present study, patients up to 16 years of age were considered pediatric, whereas those >16 years were adults. In the present study, 62.5% (50/80) of aplastic anemia cases were pediatric, and 37.5% (30/80) of the cases were adults. Paediatric patients nowadays are diagnosed earlier due to increased awareness of parents and easily available nearby diagnostic facilities. In this study, the mean of haematological parameters such as Hb, TLC, percentage of neutrophils and platelet counts was significantly lower in cases in comparison to controls (p<0.001). A previous study that was conducted earlier also displayed results in concordance with the present study^14^. The morphological evaluation of bone marrow aspiration smears, imprints, and biopsy showed that there is suppression of all three lineages along with a relative increase in bone marrow lymphocytes and plasma cells in all cases with no evidence of hemoparasites. Bone marrow cellularity was ≤ 25% in all the aplastic anemia patients and displayed replacement by fat cells. There was no significant difference noted among different severity grades for bone marrow erythroid cells, lymphocytes, or plasma cells.

The aplastic anemia patients showed significantly higher mean serum IL-6 and IL-8 levels than the controls (p<0.001). Mean IL-6 and IL-8 levels were also compared according to the severity of the disease and it was observed that their levels displayed a significant incremental trend with increased severity of aplastic anemia (p<0.001). A study performed earlier also showed similar results^11^. A previous study showed that IL-8 levels in peripheral blood were 75.0±84.4 and 296.6±305.5 in NSAA and SAA, respectively, whereas in BMA, IL-8 levels were 126.3±1.2.5 and 568.8±586.9 respectively, in NSAA and SAA patients. The presence of increased IL-8 levels in the marrow and peripheral blood is significantly correlated with the severity of the disease^13^_._ A study was done by Tang X et al. also showed that high levels of IL-8 in peripheral blood had a correlation with the severity of aplastic anemia^15^_._ Therefore, it is clear that individuals with severe and very severe cases of aplastic anemia had much higher levels of IL-6 and IL-8 in their peripheral blood than patients with the non-severe disease. Increased levels of IL-6 and IL-8 were correlated with the severity of the disease and thus seem to be crucial in aplastic anemia. This study's major drawback is that it only included a small number of cases from one institution. Future multi-institutional investigations involving more cases would be necessary. The absence of patient follow-up in our study is another limitation of the study.

## CONCLUSION

Blood serum levels of IL-6 and IL-8 were increased in aplastic anemia and showed a correlation with the severity of the disease, hence appearing to play a vital role in aplastic anemia. IL-6 and IL-8 play a key role in the immune-mediated pathophysiology of aplastic anemia. Their increasing levels are alarming signals for timely implementation of the appropriate treatment regimen to stop further progression of the disease. Early diagnosis of aplastic anemia can reduce multiple blood transfusions and pre-treatment infections, thus increasing the overall survival of patients.
